# Development and Testing of the Quality Improvement Self-efficacy Inventory

**DOI:** 10.1177/0193945921994158

**Published:** 2021-03-20

**Authors:** Marianne Baernholdt, Terry L. Jones, Colleen V. Anusiewicz, Caitlin Marley Campbell, Aoyjai Montgomery, Patricia A. Patrician

**Affiliations:** 1 University of North Carolina at Chapel Hill, Chapel Hill, NC, United States; 2 Virginia Commonwealth University, Richmond, VA, United States; 3 Center for Health Outcomes and Policy Research, School of Nursing, University of Pennsylvania, Philadelphia, PA, United States; 4 School of Nursing, University of Alabama at Birmingham, Birmingham, AL, United States; 5 School of Nursing, University of Alabama at Birmingham, Birmingham, AL, United States; 6 School of Nursing, University of Alabama at Birmingham, Birmingham, AL, United States

**Keywords:** quality improvement (QI), Quality and Safety Education for Nurses, QSEN, Quality Improvement Self-Efficacy Inventory (QISEI), nursing work environment

## Abstract

Quality improvement is paramount for patient safety. Leading change for quality improvement requires nurses with knowledge and skills beyond the clinical management of patients. In this study, staff nurses working in hospitals throughout Alabama were asked via an online survey to rate their quality improvement knowledge and skills using the new 10-item Quality Improvement Self-Efficacy Inventory (QISEI) and their perceptions of the nursing work environment using the Practice Environment Scale of the Nursing Work Index. Nurses (*N* = 886) rated the basic quality improvement items higher than the more advanced items. Several nurse characteristics and the nursing work environment were associated with nurses’ ratings of their quality improvement knowledge and skills. Educators and administrators in health care organizations can use QISEI to gauge their nurses’ knowledge and skills and then develop continuous professional development opportunities aimed at improving quality and safety competencies.

At the beginning of the 21st century, the Institute of Medicine concluded that faulty system design and poor system performance are primary root causes for patient safety incidents, which requires action (Institute of Medicine [IOM], 2001). Best practices in system redesign necessitate significant involvement of frontline health workers because of their tacit knowledge of the system. Consequently, registered nurses (RNs) are acknowledged as germane to and charged with leading transformational change in health care (IOM, 2010). Leading change for quality improvement (QI) requires knowledge and skills beyond the clinical management of patients. Such skills include system thinking; performance measurement; data management; designing, implementing, and evaluating small tests of change; and human factors engineering. To guide education in knowledge needed to change the health care system, including QI, the IOM released two reports outlining core competencies for the health professions (IOM, 2003, 2010). For nursing, these core competencies focused primarily on pre-licensure education guided by the Quality and Safety Education for Nurses (QSEN) competencies (Cronenwett et al., 2007). However, whether practicing nurses actually have the QI knowledge and skills taught in their initial education or through continuing professional development is questionable. The overall goal of this study was to examine staff nurses’ perceptions of their QI skills.

## Quality and Safety Education for Nurses Competencies

The QSEN competencies emerged from the set of five core competencies recommended by the IOM for all health professions: (a) provide patient-centered care, (b) work in interdisciplinary teams, (c) employ evidence-based practice, (d) apply QI, and (e) use informatics (IOM, 2003). From these, QSEN developed the knowledge, skills, and attitudes for six competencies: (a) patient-centered care, (b) evidence-based practice, (c) teamwork and collaboration, (d) safety, (e) QI, and (f) informatics. The QSEN competencies were first developed for nursing pre-licensure programs and later also for graduate programs (Cronenwett et al., 2007). Shortly afterwards, the QSEN essentials were embedded into the Essentials of Baccalaureate Education for Professional Nursing Practice (American Association of Colleges of Nursing, 2008).

The QSEN institute offered multiple “train the trainer” educational events for faculty to facilitate diffusion of the QSEN competencies across pre-licensure programs (Barnsteiner et al., 2013). Faculty survey data from these educational events indicated that faculty felt least prepared to teach the QI competencies and that QI competencies were less integrated into the curriculum than the other five competencies. In fact, more than 30% of schools reported that QI content was not integrated into their pre-licensure curriculum. A formal pre-licensure curriculum gap analysis supported these results. Father (2013) found that the curriculum was deficient in QI-related learning activities.

Since QSEN was implemented in 2008, studies have consistently found lower ratings of QI knowledge, skills, and attitudes compared to the other QSEN competencies across students and newly graduated RNs. In a 2008 survey of 565 graduating pre-licensure RN students from 17 U.S. nursing programs, perceived preparedness for QI competencies was rated lower than the other QSEN skills (Sullivan et al., 2009). Notably, the QI competency also was rated as less important than the other QSEN competencies. Similarly, lower perceived preparedness and importance of the QI competency were reported in newly graduated RNs (Kovner et al., 2010). In a sample of 436 newly graduated RNs, 38.6% of participants across all education levels reported that they were poorly or very poorly prepared in or had never heard of QI (Kovner et al., 2010). Comparable findings were reported in a study of 541 newly graduated hospital-based RNs from 15 states (Djukic et al., 2013a). The other QSEN competencies received higher ratings than the QI competency. Few RNs perceived themselves to be adequately prepared in the use of QI models (12%), data collection (33%), data analysis (29%), measurement (28%), project implementation (24%), data analysis and monitoring tools (16%), flowcharting processes (22%), measuring current performance (20%), assessing process gaps (15%), systematically applying QI tools and methods (19%), measuring change (17%), repeating QI activities until desired improvement is reached (14%), and monitoring sustainability (12%). Furthermore, newly graduated nurses reported low frequency of participation in QI activities (Djukic et al., 2013a). In two cohorts of early career RNs in hospitals (total *N* = 539), 21.4–48.7% reported no participation across 10 QI activities. Moreover, early career RNs reported low engagement in continuing education activities related to QI (employer-sponsored QI course, conference, or online course) (Djukic et al., 2013b). On average, over half of the RNs (*N* = 400) reported receiving no continuing professional development across all 14 QI topics queried within the previous year. More recently, Dunagan (2017) reported hospital-based RNs’ attitudes toward QI items were less positive and sometimes even negative than items for all other QSEN competencies.

Finally, after graduation, there is no central regulation of continued competency in QI. While the National Council of State Boards of Nursing issued guiding principles for the requirement for re-licensure to ensure continued competency in nursing in 2007 (Thomas et al., 2010), there is limited guidance on content and length of continuing education for QI. According to Yoder-Wise (2010), only 36 states required continuing education for re-licensure and only 1 state required content related to QI competencies (Florida requires two hours on preventing medical errors). Therefore, engagement in continuing education related to QI remains sub-optimal and up to the individual nurses and the organizations they work for.

In summary, these findings highlight several issues for QI competencies: (a) RNs are not adequately prepared in pre-licensure programs, especially RNs who graduated before dissemination of the QSEN competency; (b) RNs are not seeking or given learning opportunities to build QI competence by their employers; (c) regulatory incentives after graduation are lacking; and (d) RNs have a negative effect toward QI. The negative effect toward QI has implications for voluntary-based continuing education as RNs may not choose to develop in areas they do not enjoy or perceive as valuable. However, without frontline staff with QI knowledge, patient safety cannot improve sufficiently (Baernholdt et al., 2019). Patients and their families rely on their clinicians to deliver optimal and safe care. Therefore, it is imperative that we have ways to assess frontline staffs’ QI knowledge and skills and to detect what affects QI skills and knowledge development. In particular, nurses’ work environment is associated with both nurses’ ability to improve QI knowledge and skills and patient safety (IOM, 2004).

### Measurement of Quality Improvement Knowledge and Skills

Quality improvement competencies can be measured by direct observation in clinical settings or simulations, ratings of open-ended responses to written case studies, or self-ratings. There are several QI tools, surveys, and scales for health care professionals in general and nurses in particular. Examples of the former include the Quality Improvement Knowledge Application Tool and a later revision, that is, the Quality Improvement Knowledge Application Tool-Revised, which are tools for QI knowledge assessment of medical students by others (Singh et al., 2014). The students read a case study and answer open-ended questions. Their answers are scored using the tools in three QI subsections (aim, measure, and change). Later, the Beliefs, Attitudes, Skills, and Confidence in Quality Improvement Scale with 30 items was added to assess medical students’ self-assessment. This scale has three subscales and an excellent Cronbach *α* (0.96) (Brown et al., 2019).

For nurses, Kovner et al. (2010) developed a 35-question survey based on literature and an expert advisory group. Newly graduated nurses answered how prepared they felt in specific QI topics for both the 2010 and 2013 publications mentioned earlier (Djukic et al., 2013a). Most recently, Armstrong et al. (2017) developed a 24-item scale, the Nurses’ Attitudes and Skills around Updated Safety Concepts, based on a literature review of updated patient safety definitions and concepts. The scale consists of a 7-item subscale addressing skills and a 17-item subscale addressing attitude and was used to survey 293 RNs in seven hospitals who were part of the Collaborative Alliance for Nursing Outcomes registry. The whole scale and the two subscales had acceptable Cronbach *α* values (0.73–0.67). All of the QI tools, surveys, and scales are lengthy, with at least 24 items, none of which are based on a specific framework. Therefore, we developed the Quality Improvement Self-Efficacy Inventory or Index (QISEI), a tool that assesses nurses’ perception of their confidence related to QI. QISEI is guided by a framework and informed by literature.

### Quality Improvement Self-efficacy Inventory or Index Development

**Figure 1.**
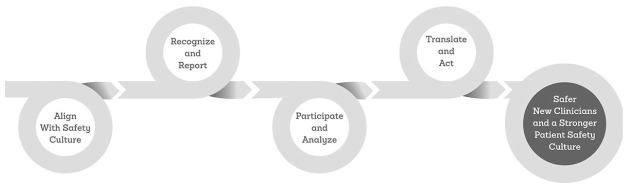
One-year journey of new clinicians to becoming a safer new clinician.

The QISEI is a 10-item inventory guided by a clinical learning environment framework to engage new clinicians in patient safety (Disch et al., 2017). The framework was developed by a national interprofessional taskforce under the National Collaborative for Improving the Clinical Learning Environment, which consists of more than 30 organizations. The taskforce issued a report with recommendations to help organizations establish supportive clinical learning environments that foster new clinicians’ abilities to become engaged in patient safety using a framework and a subsequent driver diagram. The framework was informed by literature, clinical experiences in a variety of health care settings, and basic quality and safety education requirements for each health care discipline (i.e., medicine, nursing, and pharmacy). In this framework, it is suggested that organizations support the new clinician in acquiring skills through four phases before they are competent in patient safety. The four phases are (a) align with safety culture, (b) recognize and report, (c) participate and analyze, and (d) translate and act (see Figure 1). Associated with these four phases are descriptions of behaviors for each phase. From these descriptions, we developed the 10 QISEI items. Additional literature that was used to inform the wording of the items is Kovner et al.’s (2010) survey (3 items) and QSEN (Cronenwett et al., 2007) (5 items).

Using the framework, the descriptions for each phase, and literature, we developed 10 items, which are divided into the following phases: (a) align with safety culture has two behaviors, (b) recognize and report has three behaviors, (c) participate and analyze has three behaviors, and (d) translate and act has two behaviors (see Table 1). The respondents are prompted to rate their confidence in engaging in the behaviors on a four-point scale. In contrast to a reflective scale where the items share a common concept (i.e., depression), QISEI is an example of a formative index where the components, when taken together, make up the construct, which is self-efficacy among nurses or other clinicians regarding QI (Polit & Yang, 2016). Face validity and readability of QISEI were examined in an online survey of attendees (nursing educators) at the QSEN International Forum in 2018 (*n* = 12) and a series of individual interviews of staff nurses and managers (*n* = 12) at a large academic medical center. All 24 participants provided qualitative feedback after completing the inventory. All items were deemed relevant by participants, and no recommendations for additional items were offered. For two items, examples were added to clarify the meaning of unfamiliar terms. Therefore, face validity was supported.

**Table 1.** Scores on the Practice Environment Scale of the Nursing Work Index and QISEI Instrument Ratings (*N* = 886).

Variable	*n*	Mean (SD)	Median
Practice environment scale of the nursing work index (range: 1–4)			
Nurse participation in hospital affairs (9 items)	885	2.72 (0.72)	2.78
Nursing foundations for quality of care (10 items)	885	3.08 (0.60)	3.10
Nurse manager ability, leadership, and support of nurses (5 items)	885	2.82 (0.86)	3.00
Staffing and resource adequacy (4 items)	885	2.50 (0.87)	2.50
Collegial nurse–physician relations (3 items)	885	3.07 (0.69)	3.00
Composite score	885	2.84 (0.62)	2.84
Ratings for each item on the QISEI (range: 1–4)			
1. Identifying system issues that contribute to patient safety problems	886	3.07 (0.77)	3.00
2. Applying lessons learned from mistakes of peers, teams, and self to improve patient safety	886	3.31 (0.69)	3.00
3. Communicating concerns about hazards to patients and families	886	3.23 (0.72)	3.00
4. Communicating concerns about hazard to colleagues (team)	886	3.23 (0.72)	3.00
5. Using organizational error reporting systems for near-miss and error reporting	886	2.98 (0.86)	3.00
6. Engaging in root-cause analyses when errors or near-misses occur	886	2.77 (0.90)	3.00
7. Applying tools and methods systematically to collect and analyze data for performance improvement	886	2.72 (0.90)	3.00
8. Working in a team to improve processes or systems of care as a result of errors that were reported back to your unit	886	2.91 (0.88)	3.00
9. Using national patient safety resources, initiatives, or regulations such as National Quality Forum or Institute of Health care Improvement to guide improvement initiatives on your unit	886	2.68 (0.92)	3.00
10. Repeating measurement, assessment, and applications of tools for improvement and evaluate changes until desired performance is sustained	886	2.79 (0.87)	3.00
Overall QISEI rating		2.97 (0.65)	3.00

## Purpose

The purpose of this study is threefold:

1. To evaluate the performance of QISEI among staff nurses.
2. To examine which nurse and unit characteristics were associated with overall QISEI ratings.
3. To compare ratings from nurses educated before and after QSEN was incorporated into the Essentials of Baccalaureate Education for Professional Nursing and, thus, presumably embedded into undergraduate curricula in nursing schools.

## Methods

### Design

This is a cross-sectional study using a convenience sample of state-wide nurses in Alabama. From the Alabama Board of Nursing, we purchased a roster of licensed RNs in Alabama. The roster included only names and postal addresses of the nurses. The study received IRB approval from the principal investigator’s university (IRB-300000916).

### Data Collection

In July 2018, 58,996 postcards with a survey link were mailed to RNs presumed to be working in Alabama. Upon accessing the survey, a screening question asked if the individual was a staff RN working in an acute care Alabama hospital. Only affirmative responses could proceed with the survey. The study was advertised by the Alabama State Nurses Association (ASNA), the Birmingham Regional Organization of Nursing Leaders, the Alabama Black Nurses Association, and in *The Alabama Nurse*, which is a quarterly publication provided to Alabama’s nursing community (Anusiewicz et al., in press). Additionally, reminders to participate in the study were periodically posted on ASNA and research team’s individual Facebook pages. Finally, an email was sent to all hospital Chief Nursing Officers in November 2018 asking them to remind their staff nurses about the study. Data collection ended in mid-January 2019. A total of 1,354 nurses responded to the online survey for an estimated response rate of 4.47% (Anusiewicz et al., in press).

### Measures

This study included variables for eight nurse characteristics, two unit characteristics, and QSEI ratings. The nurse characteristics such as gender (female and male), race (White, African American, and other [i.e., American Indian or Alaska Native, Asian/Pacific Islander, and other]), marital status (single or married), and children (yes/no) were included, as previous studies have found they may be associated with barriers to pursuing continuing professional development (Ikenwilo & Skåtun, 2014; Summers, 2015). Level of nursing education (Bachelor of Science in Nursing [BSN] or higher and diploma/associate degree), age, years worked as an RN, years worked in current hospital, and years worked in current unit were included, as they previously have been associated with nurses’ ratings of their work environment (Schmalenberg & Kramer, 2008). Unit characteristics included unit type (medical/surgical, intensive care, other [i.e., obstetrics, operating/recovery room, pediatrics, psychiatry, and rehabilitation]), and the nursing work environment, which was measured using the Practice Environment Scale of the Nursing Work Index. This is a 31-item scale with five subscales and a composite score (Lake, 2002). The scale has good internal consistency reliability (*α* = 0.88–0.98) (Lake & Friese, 2006). Using a four-point Likert scale, nurse respondents were asked to indicate (1 = *strongly disagree* to 4 = *strongly agree*) the degree to which each of the items are present at their current job. QISEI asked nurses to indicate if they were *not confident* = 1, *somewhat confident* = 2, *confident* = 3, or *very confident* = 4 in performing each of the QI behaviors. The individual’s inventory responses were averaged to obtain an overall QISEI rating. The higher the mean, the more confident the nurse was in performing each QI behavior.

### Data Analysis

Data were analyzed using SPSS. Participants were included only if they responded to all QISEI items. However, for other variables, the sample size may vary due to missing responses. Descriptive statistics were conducted including mean, mode, standard deviation, and range as well as frequency and percentages where appropriate. First, we examined Pearson correlations between individual QISEI items and found them to be highly correlated. Additionally, means, modes, and standard deviations were similar among the QISEI items. Therefore, only overall average ratings are included in regression models. Second, to examine associations between overall QISEI rating and nurse and unit characteristics, we conducted independent *t*-tests or one-way analysis of variance (ANOVA) (for variables with more than two categories) for each of the characteristics and the overall QISEI rating. Only variables that had correlations with *p*-values less than 0.125 (Agresti, 1996) were included in regression analyses. Third, we ran multiple regressions to determine associations between overall QISEI ratings and all explanatory variables. We included the practice environment composite score and each of the five subscales in separate analytic models due to multicollinearity among the subscales. Lastly, to test whether there were differences in ratings of QISEI after introduction of QSEN into the undergraduate curriculum, we assumed that 2010 was the first graduating year after QSEN was incorporated into the BSN Essentials to guide BSN programs. Hence, we dichotomized nurses with a BSN or a higher educational qualification (*N* = 447) into nurses with eight or fewer years of experience (i.e. indicating QSEN exposure in nursing program) and compared their QISEI ratings to nurses who had more than eight years of experience (i.e., indicating absence of QSEN exposure in their basic nursing programs) using a *t*-test.

## Results

### Descriptive

A total of 1,354 nurses responded to the online survey. The available dataset excluded hospitals that had less than three nurses responding (*n* = 125), as is the convention when including organizational variables (Lake et al., 2016, 2020; Montgomery et al., 2021). Since this is the first use of the QISEI, we did not impute missing values and, therefore, excluded nurses with any missing values on the QISEI (*n* = 343), resulting in a total sample size of 886 nurses included in this study. However, we did compare nurses with missing QISEI values to nurses who answered all items. Where data on individual QISEI items were available, we found no statistically significant differences on these items between the two groups. There were significant differences for gender and race. The group with missing QISEI data were less likely to be female (87% vs. 89.7%), had less nurses identify as White (79% vs. 82.2%), more nurses identify as African American (17.5% vs. 11.1%) and more identifying as other race (3.5% vs. 6.7%). Most respondents were married/remarried (58.1%) and reported having children (57.7%). Over half of the nurses had a BSN or higher degree (67.5%). Nurses were an average of 40 years old (*range* = 21–73) and had worked as an RN for twelve years (*range* = 0–50), in their current hospital for three years *(range* = 0–42), and on their current unit for six years *(range* = 0–60). Most respondents reported working in medical/surgical units (42.8%), though a substantial amount also reported working in intensive care units (33.2%), or another specialty unit (23.5%). The average work environment subscale scores were all 2.50 or above, (see Table 1). The overall rating and the 10 items of the QISEI were between 2.68–3.31 and mode and mean were 3 for both overall rating and all items. Nurses were most confident in applying lessons learned from mistakes of peers, teams, and self to improve patient safety (item 2) and least confident in using national patient safety resources, initiatives, or regulations to guide improvement initiatives on their unit (item 9).

### Comparison of Quality Improvement Self-efficacy Inventory Ratings and Nurse and Unit Characteristics

Quality Improvement Self-Efficacy Inventory ratings were examined according to nurse and unit characteristics. For nurse characteristics, QISEI ratings were not statistically different by gender (*t*(864) = 0.668, *p* = 0.505), education (*t*_(871)_ = −0.610, *p* = 0.542), or unit type (*F*_(2,878)_ =1.018, *p* = 0.362); however, there were statistically significant differences for QISEI ratings and marital status (*t*(861) = −2.882, *p* = 0.004), having children (*t*(866) = −4.392, *p* < 0.001), and race (*F*_(2,844)_ = 3.489, *p* = 0.031). Nurses who were married reported higher overall QISEI ratings (mean = 3.02, SD = 0.64) compared to those who were single (mean = 2.89, SD = 0.66). Nurses who had children also reported higher overall QISEI ratings (mean = 3.05, SD = 0.65) compared to those who did not have children (mean = 2.85, SD = 0.65). QSEI ratings differed between African American nurses and White nurses (mean diff = 0.18, *p* = 0.033), with African American nurses reporting higher overall QISEI ratings (mean = 3.14, SD = 0.56) compared to those who were White (mean = 2.96, SD = 0.66), while the comparison between African American and other races was not significant (mean = 2.90, SD = 0.72). Furthermore, for overall QISEI ratings, there were significant positive correlations with age, years as an RN, years in current hospital, and years on the current unit. For unit characteristics, there were significant positive correlations between overall QISEI ratings and all five subscales and composite scores of the work environment (*r* = 0.144–0.231).

### Regressions

In regression analyses, all five work environment subscales and the composite scores were significantly associated with QISEI ratings. To avoid multicollinearity, we only included years worked as an RN in the analysis (we excluded age, years in the hospital, and years worked in current unit). In Table 2, we report the work environment composite score and the subscale, Nursing Foundations for Quality of Care, as they had the highest adjusted R^2^ of all the models. The models for the other four work environment subscales (nurse participation in hospital affairs, nurse manager ability, leadership, and support of nurses, staffing and resource adequacy, and collegial nurse–physician relations) had adjusted *R*^2^ values between 0.049 and 0.087. In social and behavioral sciences, low *R*^2^ values are expected and an *R*^2^ value of 0.04 is the recommended minimum effect size (Ferguson, 2009). Overall QISEI rating was significantly associated with the work environment composite score, years worked as an RN, race, and having children (see Table 2, model 1). A one-point increase in the work environment composite score was associated with a 0.23 average increase in overall QISEI rating. A one-year increase in years worked as an RN was associated with an average increase in overall QISEI rating of 0.12. Furthermore, both African American and other compared to White race were associated with an average decrease in overall QISEI rating of 0.07, and having children was associated with a 0.09 average increase in overall QISEI rating. For the model that included the subscale, Nursing Foundations for Quality of Care, a one-point increase in the subscale was associated with a 0.27 average increase in overall QISEI rating and a one-year increase in years worked as an RN was associated with an average increase in overall QISEI rating of 0.15 (see Table 2, model 2).

**Table 2.** Multiple Linear Regression Models with Quality Improvement as an Outcome (*N* = 886).

Model 1				Model 2			
Predictors	*B* (SE)	t	*p*-Value	Predictors	*B* (SE)	t	*p*-Value
Education (BSN vs. Diploma)	0.05 (0.05)	1.33	0.185	Education (BSN vs diploma)	0.05 (0.05)	1.47	0.143
Marital status	0.03 (0.05)	0.73	0.467	Marital status	0.03 (0.05)	0.67	0.505
Race	−0.07 (0.05)	−2.01	0.044*	Race	−0.06 (0.05)	−1.57	0.118
Children	0.09 (0.06)	2.04	0.042*	Children	0.09 (0.06)	1.97	0.050
Gender	−0.01 (0.07)	−0.36	0.721	Gender	−0.00 (0.07)	−0.12	0.905
Unit	−0.05 (0.03)	−1.38	0.168	Unit	−0.04 (0.03)	−1.07	0.283
Years as RN	0.12 (0.00)	3.02	0.003*	Years as RN	0.15 (0.00)	3.66	0.000**
PES composite Score	0.23 (0.04)	6.56	0.000**	PES NFQC	0.27 (0.04)	7.60	0.000**
**ANOVA**			*p*-Value	**ANOVA**			*p*-Value
*F*_(8,747)_	9.75		0.000**	*F*_(8,747)_	11.67		0.000**
Adjusted *R*^2^	0.09			Adjusted *R*^2^	0.10		

Note: PES = Practice Environment Scale of the Nursing Work Index. PES NFQC = Nursing Foundations for Quality of Care subscale on the Practice Environment Scale of the Nursing Work Index.

* *p* < .05 and **p < .001.

### Comparison of QISEI Ratings before and after Quality and Safety Education for Nurses Implementation

Nurses with eight years or fewer of nursing experience (*n* = 275) had statically significant lower ratings of overall QISEI (mean = 2.88, SD = 0.66) compared to nurses with more than eight years of experience (*n* = 172; mean = 3.02, SD = 0.63; *p* = 0.030).

## Discussion

This study examined nurses’ ratings of QI self-efficacy components and nurse and unit characteristics that influenced these ratings. We found that ratings across the 10 items varied, and that several nurse characteristics and their work environments were associated with nurses’ ratings of their QI self-efficacy. QISEI was developed from a framework that depicts how clinicians acquire QI behaviors and skills building on knowledge as they progress through the four phases from aligning with safety culture (basic) to translation and action (advanced). Because the items of the QISEI were ordered from basic to more advanced QI behaviors and skills, we observed that nurses in this study were more confident about the basic QI behaviors and skills, as the first four items had average ratings higher than 3 compared to the ratings of items in the later part of the scale where the last six items had average ratings less than 3. The RNs in our study rated the use of national QI resources the lowest. This is similar to previous findings in newly graduated nurses where few RNs felt they were prepared to use QI models in their practice, and the majority reported no participation in QI activities (Djukic et al., 2013a, 2013b).

Achieving and sustaining QI knowledge and skills requires engagement in learning activities in pre-licensure academic programs and continuous professional development activities post licensure. Empirical inquiry into factors that influence engagement in professional development is most heavily influenced by two synergistic frameworks: self-regulated learning (Zimmerman, 1990) and self-determination theory (Ryan & Deci, 2000). Collectively, these frameworks support self-regulatory attributes and motivation profiles as influential factors underlying an RN’s decision to engage in QI knowledge and skills development. Importantly, they also assert that motivational profiles are influenced by sociocultural context.

Two sociocultural factors, that is, marital status and having children, were examined in this study. Resource-related barriers to continuous professional development are commonly reported among health care providers to include lack of time, financial costs, travel requirements, and childcare (Ikenwilo & Skåtun, 2014; Summers, 2015). Arguably, single and childless RNs might have more discretionary time and financial resources available for QI-related professional development, greater flexibility for travel, and no need for childcare. Surprisingly, RNs in this study who were married with children reported higher QI self-efficacy than single and childless RNs. Post-hoc analysis revealed that married RNs with children were older. It may be that the married RNs in this study had children past the age of needing childcare during the RNs time devoted to professional development. The higher age may also reflect the RNs who have more clinical experience and consequently more opportunity for experiential learning related to QI knowledge and skills. More research is needed to examine the influence of social-cultural factors on development of QI knowledge and skills.

Unexpectedly, we found that having a baccalaureate degree was not associated with QISEI ratings. Also, nurses who had eight years or less of nursing experience had lower ratings of QI knowledge and skills compared to their colleagues with more than eight years of experience. We expected to find that the less experienced nurses would score higher on the QISEI because they had the QSEN competencies ingrained into their pre-licensure programs, since the QSEN competencies were widely accepted as having been incorporated into undergraduate curricula nationally by 2010. These findings are similar to earlier studies of newly graduated nurses whether they graduated before or after QSEN was embedded into the Baccalaureate Essentials and, therefore, into nursing programs across the United States (Djukic et al., 2013a, 2013b; Kovner et al., 2010). Clearly, the QSEN QI competencies are still not acquired sufficiently in undergraduate programs or in the first years of practice. Several explanations are possible. First, perhaps new nurses are so overwhelmed with becoming proficient in their clinical skills that QI competencies are forgotten, not prioritized, or not acquired in those early years. Second, if organizations do not have structures and processes that require nurses to be active in QI in place, it is difficult for staff nurses to acquire QI skills on the job. The findings that nurses with more experience rated their QI self-efficacy higher is echoed in the regression results where we found that years as an RN was associated with higher overall QISEI ratings.

Consistent with reviews on the importance of better work environments for quality and safety (Copanitsanou et al., 2017; Halm, 2019; Lake et al., 2019; Lee & Scott, 2018; Nascimento & Jesus, 2020; Stalpers et al., 2015; Wei et al., 2018), we found higher ratings on both the practice environment composite score, which is an overall measure of the nursing work environment, and the subscale, Nursing Foundations for Quality of Care. Additionally, both were significantly associated with higher QISEI ratings. Optimal nursing work environments are those in which nurses have enough staff and other resources to effectively monitor patients as well as the knowledge, skill, and accountability for safe patient care (American Association of Colleges of Nursing, n.d.). Furthermore, these environments provide opportunities for continuing professional development for nurses, encourage teamwork and good relationships with other health care members, and emphasize involvement and engagement in organizational policies that affect patient care.

To the best of our knowledge, this is the first study that found the Nursing Foundations for Quality of Care subscale to be associated with ratings of QI knowledge and skills.This is not surprising considering the subscale includes elements that would enhance the development of QI knowledge and skills, namely the presence of performance improvement, continuing education and preceptorships, high-performance standards, and competent staff (Lake, 2002). Other studies have found significant relationships between this subscale and outcomes. Higher scores on the Nursing Foundations for Quality of Care subscale were associated with near-misses or error interception practices, which were in turn associated with fewer medication errors among medical/surgical nurses (Flynn et al., 2012). In military hospitals, the subscale was associated with higher ratings of patient experiences of nursing care, but not adverse events (Swiger et al., 2018). Both study findings emphasize the importance of RNs’ QI knowledge and skills. Our findings and these two studies underscore that although most research has included the work environment composite score to gauge nursing work environments, differential analysis of the subscales that represent related but distinct components of the nursing work environment continues to be relevant as we increase our understanding of how to best improve quality and safety of health care delivery. In summary, our findings suggest there is a need to further explore the relationships between basic education content (QSEN), years of RN experience, work environments and other organizational components, and the development of QI knowledge and skills proficiency.

Our study had several limitations. Due to the inability to obtain email addresses of RNs currently working in Alabama hospitals from the Alabama State Board of Nursing and limited funds for follow-up postal surveys, the response rate was low, although a post-hoc power analysis found that the sample size of 866 was sufficient to achieve power. Furthermore, we did find that the sample of nurses who had missing values on QISEI had more participants identifying as female and White compared to participants with no missing values. However, when compared to the 2016 Alabama’s RN Workforce Demographic survey (Alabama Board of Nursing, 2017), our sample is representative of the Alabama nursing workforce, other than level of education (over half of the Alabama nursing workforce has an Associate Degree in Nursing in the 2016 survey). Lastly, because this was a cross-sectional analysis, the resulting associations cannot be determined as causative.

In conclusion, quality and safety competencies must remain important to educators and administrators in health care organizations if patient safety is to be improved. Academic and health care leaders can use the QISEI to gather information about the level of QI knowledge and skills of their respective faculty or staff and use the results to develop organizational changes and continuous professional development opportunities aimed at improving and incorporating QI competencies into nursing education and practice. However, in order to effectively incorporate QI knowledge and skills into routine nursing practice, there must be organizational structures and processes that support and encourage continuous learning in place.
